# 1-[5-Acetyl-4-(4-bromo­phen­yl)-2,6-dimethyl-1,4-dihydro­pyridin-3-yl]ethanone monohydrate

**DOI:** 10.1107/S1600536810006124

**Published:** 2010-02-20

**Authors:** Palakshi B. Reddy, V. Vijayakumar, S. Sarveswari, T. Narasimhamurthy, Edward R. T. Tiekink

**Affiliations:** a Organic Chemistry Division, School of Advanced Sciences, VIT University, India; b Materials Research Centre, Indian Institute of Science, Bengaluru 560 012, India; c Department of Chemistry, University of Malaya, 50603 Kuala Lumpur, Malaysia

## Abstract

The 1,4-dihydro­pyridine ring in the title hydrate, C_17_H_18_BrNO_2_·H_2_O, has a flattened-boat conformation, and the benzene ring is occupies a position orthogonal to this [dihedral angle: 82.19 (16)°]. In the crystal packing, supra­molecular arrays mediated by N—H⋯O_water_ and O_water_—H⋯O_carbon­yl_ hydrogen bonding are formed in the *bc* plane. A highly disordered solvent mol­ecule is present within a mol­ecular cavity defined by the organic and water mol­ecules. Its contribution to the electron density was removed from the observed data in the final cycles of refinement and the formula, mol­ecular weight and density are given without taking into account the contribution of the solvent mol­ecule.

## Related literature

For background to the pharmacological potential of Hantzsch 4-dihydro­pyridines, see: Gaudio *et al.* (1994 ▶); Böcker & Guengerich (1986 ▶); Gordeev *et al.* (1996 ▶); Sunkel *et al.* (1992 ▶); Vo *et al.* (1995 ▶); Cooper *et al.* (1992 ▶). For the synthesis, see: Rathore *et al.* (2009 ▶). For a related structure, see: de Armas *et al.* (2000 ▶). For additional geometric analysis, see: Cremer & Pople, (1975 ▶).
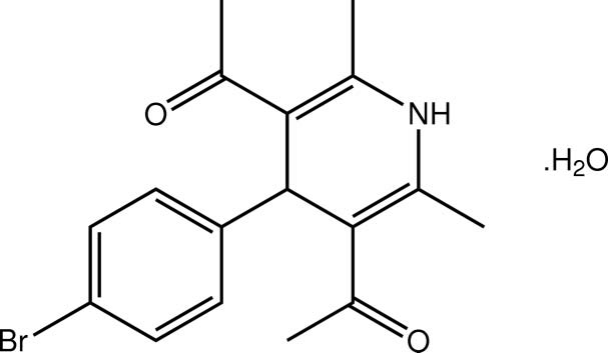


## Experimental

### 

#### Crystal data

C_17_H_18_BrNO_2_·H_2_O
*M* *_r_* = 366.25
Monoclinic, 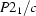

*a* = 13.5236 (3) Å
*b* = 10.3866 (2) Å
*c* = 15.0939 (3) Å
β = 102.112 (1)°
*V* = 2072.96 (7) Å^3^
*Z* = 4
Mo *K*α radiation
μ = 1.99 mm^−1^
*T* = 293 K
0.21 × 0.11 × 0.10 mm

#### Data collection

Bruker SMART APEX CCD diffractometer
Absorption correction: multi-scan (*SADABS*; Bruker, 1998 ▶) *T*_min_ = 0.768, *T*_max_ = 0.819
27847 measured reflections
3658 independent reflections
2611 reflections with *I* > 2σ(*I*)
*R*_int_ = 0.032

#### Refinement

*R*[*F* ^2^ > 2σ(*F* ^2^)] = 0.049
*wR*(*F* ^2^) = 0.147
*S* = 1.08
3658 reflections
212 parameters
4 restraints
H atoms treated by a mixture of independent and constrained refinement
Δρ_max_ = 0.56 e Å^−3^
Δρ_min_ = −0.75 e Å^−3^

### 

Data collection: *SMART* (Bruker, 2001 ▶); cell refinement: *SAINT* (Bruker, 2001 ▶); data reduction: *SAINT*; program(s) used to solve structure: *SHELXS97* (Sheldrick, 2008 ▶); program(s) used to refine structure: *SHELXL97* (Sheldrick, 2008 ▶) and *PLATON* (Spek, 2009 ▶); molecular graphics: *ORTEP-3* (Farrugia, 1997 ▶) and *DIAMOND* (Brandenburg, 2006 ▶); software used to prepare material for publication: *publCIF* (Westrip, 2010 ▶).

## Supplementary Material

Crystal structure: contains datablocks general, I. DOI: 10.1107/S1600536810006124/hg2647sup1.cif

Structure factors: contains datablocks I. DOI: 10.1107/S1600536810006124/hg2647Isup2.hkl

Additional supplementary materials:  crystallographic information; 3D view; checkCIF report

## Figures and Tables

**Table 1:** Hydrogen-bond geometry (Å, °)

*D*—H⋯*A*	*D*—H	H⋯*A*	*D*⋯*A*	*D*—H⋯*A*
N1—H1n⋯O1w	0.88 (1)	2.03 (1)	2.904 (3)	174 (2)
O1*W*—H1*w*⋯O1^i^	0.84 (2)	1.92 (3)	2.754 (3)	174 (4)
O1*W*—H2*w*⋯O2^ii^	0.84 (2)	1.96 (2)	2.778 (3)	166 (2)

Symmetry codes: (i) 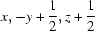; (ii) 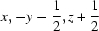.

**Fig. 1.**
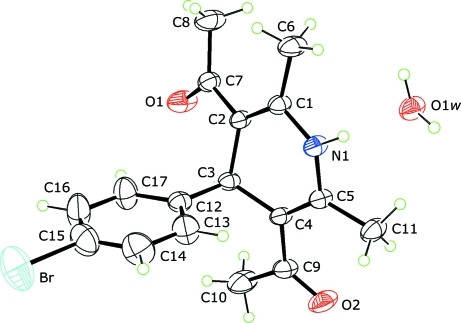
The molecular structure of (I) showing the atom-labelling scheme and displacement ellipsoids at the 35% probability level.

**Fig. 2.**
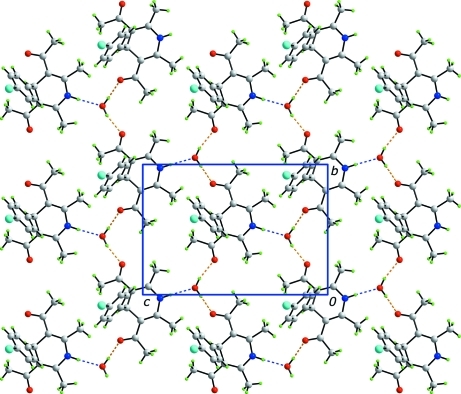
A view of a supramolecular array formed in the bc plane of (I). The N–H···O and O–H···O hydrogen bonds are shown as blue and orange dashed lines, respectively. Colour code: Br, cyan; O, red; N, blue; C, grey; and H, green.

**Fig. 3.**
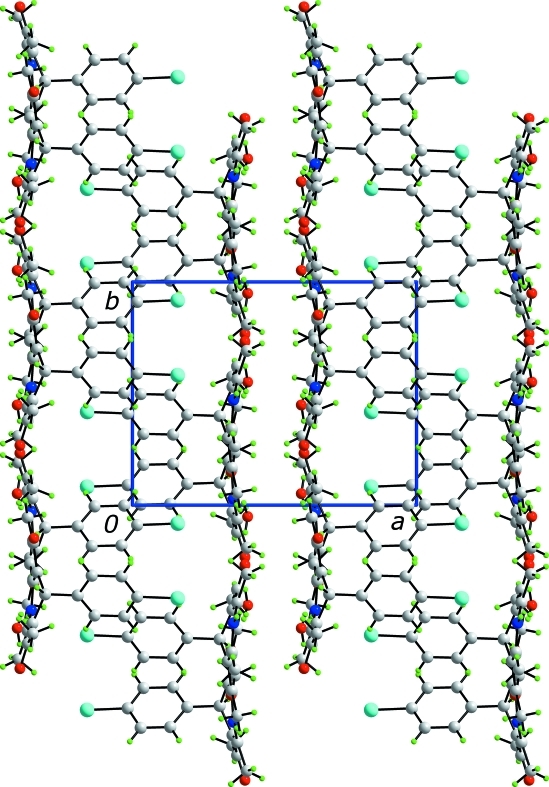
A view in projection down the c axis highlighting the sandwich-like packing along the a axis in (I). Colour code: Br, cyan; O, red; N, blue; C, grey; and H, green.

## supplementary crystallographic information

### Comment

Biologically active compounds based on Hantzsch 1,4-dihydropyridines (DHPs) have been demonstrated to possess a range of pharmaceutical properties, such as vasodilator, antihypertensive, bronchodilator, heptaprotective, anti-tumour, anti-mutagenic, geroprotective and anti-diabetic agents (Gaudio *et al.*, 1994). For example, calcium channel blockers Nifedipine, Nitrendipine and Nimodipine have found commercial utility (Böcker & Guengerich, 1986; Gordeev *et al.*, 1996). Various DHP-based calcium antagonists have been introduced for the treatment of congestive heart failure (Sunkel *et al.*, 1992; Vo *et al.*, 1995). Finally, a number of DHPs having anti-aggregatory activity of platelet are known (Cooper *et al.*, 1992). In continuation of study investigating crystal packing motifs of these compounds (Rathore *et al.* (2009), the title hydrate, (I), was investigated.

The molecular structures of the components of (I) are shown in Fig. 1. The 1,4-dihydropyridine ring in (I) has a flattened-boat conformation with the N1 and C3 atoms lying above the plane defined by the C1,C2,C4 and C5 atoms. This assignments is quantified by the ring puckering parameters (Cremer & Pople, 1975): Q = 0.312 (3) Å, θ = 72.0 (6) °, and φ_2_ = 175.4 (5) °. The aryl ring is orthogonal to the 1,4-dihydropyridine ring with a dihedral angle between their respective least-squares planes of 82.19 (16) °. The observed conformation in (I) is entirely consistent with those found for the two closely related aryl derivatives of (I), *i*.*e*. with PhNO_2_-3 (Rathore *et al.*, (2009) and with PhOH-4, as the monohydrate (de Armas *et al.*, 2000). The difference between the structures relate to the relative disposition of the acetyl groups. In each case, these are essentially co-planar with the 1,4-dihydropyridine ring and in the PhNO_2_-3 derivative (Rathore *et al.*, (2009), both carbonyl atoms are orientated away from the amine group whereas in (I) and in PhOH-4 monohydrate (de Armas *et al.*, 2000), one is orientated towards the amine group.

The crystal packing features N–H···O_water_ and O_water_–*H*···O_carbonyl_ hydrogen bonding, Table 1. These link the molecules into a layer in the *bc* plane, Fig. 2, with all the aryl rings being orientated to one side of the plane for each layer. Pairs of layers interdigitate to form sandwich structure, Fig. 3.

### Experimental

3,5-Diacetyl-2,6-dimethyl-1,4-dihydro-4-(4-bromophenyl)-pyridine was prepared according to Hantzsch pyridine synthesis (Rathore *et al.*, 2009). 4-Bromobenzaldehyde (10 mmol), acetylacetone (20 mmol) and ammonium acetate (10 mmol) were taken in a 1:2:1 mole ratio along with ethanol (20 ml) as solvent in a RB-flask and refluxed over a steam-bath until the colour of the solution changed to red-orange (approximately 2 h). The solution was kept under ice-cold conditions in order to precipitate the solid product. This was extracted using diethyl ether and then excess solvent was distilled off. The purity of the crude product was checked through TLC and recrystallized using mixture of acetone and diethyl ether (3:1); Yield: 85%; m.pt. 382 K. Crystals were grown from an acetone and ether (3:1) solution over three days. IR (KBr): ν(N—H) 3358, ν(Ar—H) 3062, ν(C═O) 1678, ν(C–Br) 744 cm^-1^.

### Refinement

The C-bound H atoms were geometrically placed (C–H = 0.93–0.96 Å) and refined as riding with *U_iso_*(H) = 1.2–1.5*U_eq_*(C). The remaining H were located from a difference map and refined with O–H = 0.840±0.001 (with H1w···H2w = 1.39±0.01) and N–H = 0.880±0.001, and with *U_iso_*(H) = n*U_eq_*(parent atom), with n = 1.5 for O and n = 1.2 for N. Unresolved disordered solvent was evident in the final cycles of the refinement. This was modelled with the SQUEEZE option in PLATON (Spek, 2009); the solvent cavity had volume 251 Å^3^. In the final cycles of refinement, this contribution to the electron density was removed from the observed data. The density, the F(000) value, the molecular weight, and the formula are given without taking into account the contribution of the solvent molecule(s). The structure factor programme detects differences in *R* values. These discrepancies arise as the structure factor checking program does not take into account the SQUEEZE procedure applied to the data, as explained in the refinement section, and appended at the end of the CIF.

### Crystal data

**Table:**

C_17_H_18_BrNO_2_·H_2_O	*F*(000) = 752
*M**_r_* = 366.25	*D*_x_ = 1.174 Mg m^−^^3^
Monoclinic, *P*2_1_/*c*	Mo *K*α radiation, λ = 0.71073 Å
Hall symbol: -P 2ybc	Cell parameters from 7408 reflections
*a* = 13.5236 (3) Å	θ = 2.4–22.7°
*b* = 10.3866 (2) Å	µ = 1.99 mm^−^^1^
*c* = 15.0939 (3) Å	*T* = 293 K
β = 102.112 (1)°	Block, colourless
*V* = 2072.96 (7) Å^3^	0.21 × 0.11 × 0.10 mm
*Z* = 4	

### Data collection

**Table:**

Bruker SMART APEX CCD diffractometer	3658 independent reflections
Radiation source: fine-focus sealed tube	2611 reflections with *I* > 2σ(*I*)
graphite	*R*_int_ = 0.032
ω scans	θ_max_ = 25.0°, θ_min_ = 1.5°
Absorption correction: multi-scan (*SADABS*; Bruker, 1998)	*h* = −16→15
*T*_min_ = 0.768, *T*_max_ = 0.819	*k* = 0→12
27847 measured reflections	*l* = 0→17

### Refinement

**Table:**

Refinement on *F*^2^	Primary atom site location: structure-invariant direct methods
Least-squares matrix: full	Secondary atom site location: difference Fourier map
*R*[*F*^2^ > 2σ(*F*^2^)] = 0.049	Hydrogen site location: inferred from neighbouring sites
*wR*(*F*^2^) = 0.147	H atoms treated by a mixture of independent and constrained refinement
*S* = 1.08	*w* = 1/[σ^2^(*F*_o_^2^) + (0.071*P*)^2^ + 1.0899*P*] where *P* = (*F*_o_^2^ + 2*F*_c_^2^)/3
3658 reflections	(Δ/σ)_max_ = 0.001
212 parameters	Δρ_max_ = 0.56 e Å^−^^3^
4 restraints	Δρ_min_ = −0.75 e Å^−^^3^

### Special details

**Table:**

Geometry. All s.u.'s (except the s.u. in the dihedral angle between two l.s. planes) are estimated using the full covariance matrix. The cell s.u.'s are taken into account individually in the estimation of s.u.'s in distances, angles and torsion angles; correlations between s.u.'s in cell parameters are only used when they are defined by crystal symmetry. An approximate (isotropic) treatment of cell s.u.'s is used for estimating s.u.'s involving l.s. planes.
Refinement. Refinement of *F*^2^ against ALL reflections. The weighted *R*-factor wR and goodness of fit *S* are based on *F*^2^, conventional *R*-factors *R* are based on *F*, with *F* set to zero for negative *F*^2^. The threshold expression of *F*^2^ > 2σ(*F*^2^) is used only for calculating *R*-factors(gt) etc. and is not relevant to the choice of reflections for refinement. *R*-factors based on *F*^2^ are statistically about twice as large as those based on *F*, and *R*- factors based on ALL data will be even larger.

### Fractional atomic coordinates and isotropic or equivalent isotropic displacement parameters (Å^2^)

**Table:**

	*x*	*y*	*z*	*U*_iso_*/*U*_eq_	
Br	−0.15936 (3)	0.08244 (7)	0.77808 (4)	0.1238 (3)	
O1	0.3468 (2)	0.35143 (19)	0.87926 (13)	0.0640 (6)	
O2	0.3961 (2)	−0.2324 (2)	0.88848 (14)	0.0691 (7)	
N1	0.35579 (19)	0.0281 (2)	1.09364 (14)	0.0423 (5)	
H1N	0.367 (2)	0.001 (3)	1.1502 (7)	0.051*	
C1	0.3493 (2)	0.1585 (2)	1.07651 (16)	0.0388 (6)	
C2	0.33562 (19)	0.1993 (2)	0.98949 (16)	0.0350 (6)	
C3	0.3071 (2)	0.1007 (2)	0.91317 (16)	0.0356 (6)	
H3	0.3358	0.1303	0.8623	0.043*	
C4	0.3524 (2)	−0.0305 (2)	0.94161 (16)	0.0365 (6)	
C5	0.3691 (2)	−0.0629 (2)	1.03099 (17)	0.0389 (6)	
C6	0.3600 (3)	0.2362 (3)	1.16203 (18)	0.0559 (8)	
H6A	0.4253	0.2768	1.1752	0.084*	
H6B	0.3535	0.1805	1.2113	0.084*	
H6C	0.3082	0.3008	1.1542	0.084*	
C7	0.3489 (2)	0.3316 (2)	0.96005 (17)	0.0425 (6)	
C8	0.3688 (3)	0.4445 (3)	1.0225 (2)	0.0683 (10)	
H8A	0.3782	0.5201	0.9886	0.102*	
H8B	0.4287	0.4289	1.0681	0.102*	
H8C	0.3123	0.4570	1.0508	0.102*	
C9	0.3687 (2)	−0.1214 (3)	0.87207 (19)	0.0463 (7)	
C10	0.3516 (3)	−0.0759 (3)	0.7756 (2)	0.0723 (11)	
H10A	0.3597	−0.1469	0.7370	0.108*	
H10B	0.3997	−0.0099	0.7705	0.108*	
H10C	0.2843	−0.0420	0.7577	0.108*	
C11	0.4025 (3)	−0.1911 (3)	1.07260 (19)	0.0558 (8)	
H11A	0.3465	−0.2500	1.0612	0.084*	
H11B	0.4262	−0.1811	1.1368	0.084*	
H11C	0.4561	−0.2243	1.0464	0.084*	
C12	0.1926 (2)	0.0954 (3)	0.88046 (17)	0.0417 (6)	
C13	0.1332 (2)	0.0057 (3)	0.9125 (2)	0.0638 (9)	
H13	0.1640	−0.0542	0.9553	0.077*	
C14	0.0291 (3)	0.0023 (4)	0.8828 (3)	0.0778 (11)	
H14	−0.0092	−0.0592	0.9054	0.093*	
C15	−0.0166 (3)	0.0895 (4)	0.8205 (3)	0.0766 (10)	
C16	0.0384 (3)	0.1807 (4)	0.7877 (3)	0.0858 (12)	
H16	0.0063	0.2406	0.7456	0.103*	
C17	0.1432 (3)	0.1839 (4)	0.8176 (2)	0.0686 (9)	
H17	0.1805	0.2465	0.7951	0.082*	
O1W	0.40559 (17)	−0.05147 (18)	1.28219 (12)	0.0510 (5)	
H1w	0.392 (3)	0.0100 (17)	1.3139 (18)	0.076*	
H2w	0.397 (3)	−0.1227 (12)	1.3060 (19)	0.076*	

### Atomic displacement parameters (Å^2^)

**Table:**

	*U*^11^	*U*^22^	*U*^33^	*U*^12^	*U*^13^	*U*^23^
Br	0.0512 (3)	0.1686 (6)	0.1423 (5)	−0.0066 (3)	−0.0009 (3)	0.0017 (4)
O1	0.1159 (19)	0.0375 (11)	0.0411 (11)	−0.0092 (11)	0.0222 (11)	0.0047 (9)
O2	0.117 (2)	0.0393 (12)	0.0529 (12)	0.0149 (12)	0.0218 (12)	−0.0105 (10)
N1	0.0692 (15)	0.0318 (12)	0.0288 (11)	0.0016 (11)	0.0171 (11)	0.0033 (9)
C1	0.0527 (16)	0.0324 (14)	0.0334 (13)	−0.0002 (12)	0.0141 (11)	−0.0015 (11)
C2	0.0460 (14)	0.0276 (13)	0.0329 (13)	0.0004 (11)	0.0114 (11)	−0.0029 (10)
C3	0.0488 (15)	0.0305 (13)	0.0289 (12)	−0.0032 (11)	0.0115 (11)	−0.0018 (10)
C4	0.0500 (15)	0.0261 (13)	0.0345 (13)	−0.0033 (11)	0.0112 (11)	−0.0032 (10)
C5	0.0489 (15)	0.0315 (14)	0.0384 (14)	−0.0017 (11)	0.0139 (12)	−0.0024 (11)
C6	0.094 (2)	0.0406 (16)	0.0359 (15)	0.0006 (16)	0.0212 (15)	−0.0059 (12)
C7	0.0553 (16)	0.0337 (14)	0.0383 (15)	0.0002 (12)	0.0095 (12)	−0.0004 (11)
C8	0.119 (3)	0.0339 (16)	0.0524 (19)	−0.0096 (17)	0.019 (2)	−0.0023 (14)
C9	0.0599 (18)	0.0373 (16)	0.0431 (15)	−0.0024 (13)	0.0142 (13)	−0.0079 (12)
C10	0.128 (4)	0.053 (2)	0.0389 (17)	0.0131 (19)	0.026 (2)	−0.0095 (14)
C11	0.089 (2)	0.0354 (15)	0.0454 (16)	0.0085 (15)	0.0189 (16)	0.0056 (12)
C12	0.0522 (16)	0.0400 (14)	0.0342 (13)	−0.0012 (13)	0.0123 (12)	−0.0037 (11)
C13	0.057 (2)	0.064 (2)	0.069 (2)	−0.0095 (16)	0.0116 (16)	0.0129 (17)
C14	0.061 (2)	0.088 (3)	0.087 (3)	−0.019 (2)	0.020 (2)	0.005 (2)
C15	0.0472 (19)	0.097 (3)	0.082 (3)	−0.005 (2)	0.0069 (18)	−0.005 (2)
C16	0.061 (2)	0.093 (3)	0.093 (3)	0.011 (2)	−0.005 (2)	0.028 (2)
C17	0.059 (2)	0.076 (2)	0.067 (2)	−0.0011 (18)	0.0042 (16)	0.0236 (18)
O1w	0.0796 (15)	0.0376 (10)	0.0379 (11)	0.0059 (10)	0.0170 (10)	0.0048 (8)

### Geometric parameters (Å, °)

**Table:**

Br—C15	1.904 (4)	C8—H8B	0.9600
O1—C7	1.231 (3)	C8—H8C	0.9600
O2—C9	1.221 (3)	C9—C10	1.501 (4)
N1—C5	1.375 (3)	C10—H10A	0.9600
N1—C1	1.378 (3)	C10—H10B	0.9600
N1—H1n	0.881 (14)	C10—H10C	0.9600
C1—C2	1.355 (3)	C11—H11A	0.9600
C1—C6	1.503 (4)	C11—H11B	0.9600
C2—C7	1.467 (4)	C11—H11C	0.9600
C2—C3	1.530 (3)	C12—C13	1.383 (4)
C3—C4	1.519 (3)	C12—C17	1.388 (4)
C3—C12	1.523 (4)	C13—C14	1.385 (5)
C3—H3	0.9800	C13—H13	0.9300
C4—C5	1.363 (4)	C14—C15	1.358 (6)
C4—C9	1.462 (4)	C14—H14	0.9300
C5—C11	1.501 (4)	C15—C16	1.360 (6)
C6—H6A	0.9600	C16—C17	1.394 (5)
C6—H6B	0.9600	C16—H16	0.9300
C6—H6C	0.9600	C17—H17	0.9300
C7—C8	1.492 (4)	O1w—H1w	0.84 (2)
C8—H8A	0.9600	O1w—H2w	0.841 (18)
			
C5—N1—C1	124.0 (2)	H8B—C8—H8C	109.5
C5—N1—H1N	115 (2)	O2—C9—C4	123.3 (3)
C1—N1—H1N	119 (2)	O2—C9—C10	118.2 (2)
C2—C1—N1	118.7 (2)	C4—C9—C10	118.5 (2)
C2—C1—C6	129.3 (2)	C9—C10—H10A	109.5
N1—C1—C6	112.0 (2)	C9—C10—H10B	109.5
C1—C2—C7	125.9 (2)	H10A—C10—H10B	109.5
C1—C2—C3	118.8 (2)	C9—C10—H10C	109.5
C7—C2—C3	115.3 (2)	H10A—C10—H10C	109.5
C4—C3—C12	112.4 (2)	H10B—C10—H10C	109.5
C4—C3—C2	111.4 (2)	C5—C11—H11A	109.5
C12—C3—C2	110.3 (2)	C5—C11—H11B	109.5
C4—C3—H3	107.5	H11A—C11—H11B	109.5
C12—C3—H3	107.5	C5—C11—H11C	109.5
C2—C3—H3	107.5	H11A—C11—H11C	109.5
C5—C4—C9	122.2 (2)	H11B—C11—H11C	109.5
C5—C4—C3	118.3 (2)	C13—C12—C17	116.9 (3)
C9—C4—C3	119.3 (2)	C13—C12—C3	122.5 (3)
C4—C5—N1	119.5 (2)	C17—C12—C3	120.6 (3)
C4—C5—C11	127.3 (2)	C12—C13—C14	122.0 (3)
N1—C5—C11	113.2 (2)	C12—C13—H13	119.0
C1—C6—H6A	109.5	C14—C13—H13	119.0
C1—C6—H6B	109.5	C15—C14—C13	119.4 (3)
H6A—C6—H6B	109.5	C15—C14—H14	120.3
C1—C6—H6C	109.5	C13—C14—H14	120.3
H6A—C6—H6C	109.5	C14—C15—C16	120.8 (3)
H6B—C6—H6C	109.5	C14—C15—Br	119.3 (3)
O1—C7—C2	118.6 (2)	C16—C15—Br	119.9 (3)
O1—C7—C8	117.2 (2)	C15—C16—C17	119.7 (4)
C2—C7—C8	124.2 (2)	C15—C16—H16	120.1
C7—C8—H8A	109.5	C17—C16—H16	120.1
C7—C8—H8B	109.5	C12—C17—C16	121.1 (3)
H8A—C8—H8B	109.5	C12—C17—H17	119.5
C7—C8—H8C	109.5	C16—C17—H17	119.5
H8A—C8—H8C	109.5	H1w—O1w—H2w	111 (3)
			
C5—N1—C1—C2	13.2 (4)	C3—C2—C7—O1	−7.3 (4)
C5—N1—C1—C6	−166.2 (3)	C1—C2—C7—C8	−7.1 (5)
N1—C1—C2—C7	−165.8 (3)	C3—C2—C7—C8	175.0 (3)
C6—C1—C2—C7	13.5 (5)	C5—C4—C9—O2	1.6 (5)
N1—C1—C2—C3	12.1 (4)	C3—C4—C9—O2	−173.2 (3)
C6—C1—C2—C3	−168.6 (3)	C5—C4—C9—C10	−178.1 (3)
C1—C2—C3—C4	−31.5 (3)	C3—C4—C9—C10	7.2 (4)
C7—C2—C3—C4	146.5 (2)	C4—C3—C12—C13	29.6 (3)
C1—C2—C3—C12	94.1 (3)	C2—C3—C12—C13	−95.5 (3)
C7—C2—C3—C12	−87.9 (3)	C4—C3—C12—C17	−151.8 (3)
C12—C3—C4—C5	−95.4 (3)	C2—C3—C12—C17	83.1 (3)
C2—C3—C4—C5	29.0 (3)	C17—C12—C13—C14	0.9 (5)
C12—C3—C4—C9	79.5 (3)	C3—C12—C13—C14	179.5 (3)
C2—C3—C4—C9	−156.1 (2)	C12—C13—C14—C15	−0.1 (6)
C9—C4—C5—N1	177.7 (3)	C13—C14—C15—C16	−0.7 (6)
C3—C4—C5—N1	−7.5 (4)	C13—C14—C15—Br	178.7 (3)
C9—C4—C5—C11	−1.8 (5)	C14—C15—C16—C17	0.7 (7)
C3—C4—C5—C11	173.0 (3)	Br—C15—C16—C17	−178.7 (3)
C1—N1—C5—C4	−15.7 (4)	C13—C12—C17—C16	−0.8 (5)
C1—N1—C5—C11	163.9 (3)	C3—C12—C17—C16	−179.5 (3)
C1—C2—C7—O1	170.6 (3)	C15—C16—C17—C12	0.0 (6)

### Hydrogen-bond geometry (Å, °)

**Table:**

*D*—H···*A*	*D*—H	H···*A*	*D*···*A*	*D*—H···*A*
N1—H1n···O1w	0.881 (14)	2.025 (13)	2.904 (3)	174 (2)
O1W—H1w···O1^i^	0.84 (2)	1.92 (3)	2.754 (3)	174 (4)
O1W—H2w···O2^ii^	0.84 (2)	1.96 (2)	2.778 (3)	166 (2)

Symmetry codes:  (i) *x*, −*y*+1/2, *z*+1/2; (ii) *x*, −*y*−1/2, *z*+1/2.
